# Demographic Profile and Prescribing Patterns of Anti-epileptic Drugs in Indian Epilepsy Patients: Electronic Medical Record-Based Nation-Wide Retrospective Cohort Study

**DOI:** 10.7759/cureus.23676

**Published:** 2022-03-31

**Authors:** Atmaram Bansal, Shalin Shah, Shiva Kumar, Amit Haldar, Madhusudan B.K, Smita Brahma, Kumar Gaurav, Colette Pinto, Amey Mane, Snehal Shah

**Affiliations:** 1 Epilepsy Programme, Institute of Neurosciences, Medanta Hospital, Gurugram, IND; 2 Neurology, Sheth VS (Vadilal Sarabhai) Hospital and NHL (Nathiba Hargovandas Lakhmichand) Municipal Medical College, Ahmedabad, IND; 3 Neurology, Sakra World Hospital, Bangalore, IND; 4 Neurology, Fortis Hospital, Kolkata, IND; 5 Neurology, BGS Global Hospitals, Bengaluru, IND; 6 Medical Affairs, Dr. Reddy’s Laboratories Ltd., Hyderabad, IND; 7 Clinical Research and Medical Affairs, Dr. Reddy’s Laboratories Ltd., Hyderabad, IND; 8 Insights, HealthPlix Technologies, Bengaluru, IND

**Keywords:** real world, epilepsy, demographics, treatment patterns, india

## Abstract

The aim of this study was to provide real-world data on clinical characteristics, risk factors, and treatment patterns in Indian patients with epilepsy. Electronic medical record (EMR) data of patients diagnosed with epilepsy between January 2001 and December 2019, which included demographics, diagnosis, anti-epileptic drug usage, and underlying risk factors were evaluated. The majority of patients were between the age group of 18 and 55 years (n=3,186), with males accounting for 62% and the remaining 38% being females. Further, the most common comorbidity was hypertension (23.3%, n=1,470), followed by diabetes mellitus (10.8%, n=683) and depression (9.4%, n=597). The most prevalent form of epilepsy was focal epilepsy (n=5,141 81.4%), followed by generalized epilepsy (n=601). Focal epilepsy was most prevalent in males (62%, n=3,167) and most common in the age group of 18-55 years (50.3%, n=2588). Anti-epileptic drug (AED) usage data from 6,318 patients showed that the most commonly prescribed AED alone or in combination for both focal and generalized epilepsy was levetiracetam (41.8%, n= 2645). Data collected from this study are aligned but do not completely agree with the Guidelines for the Management of Epilepsy in India (GEMIND). This affirms treatment initiation with AED monotherapy; however, the treatment choices do not necessarily follow the recommended guidelines to select conventional AEDs, at low strengths, at initiation.

## Introduction

Epilepsy or seizure disorder is a chronic neurological condition characterized by recurrent unprovoked seizures, which are brief episodes of involuntary movement that may be focal/partial (involving only a part of the body) or generalized (involving the entire body), sometimes accompanied by loss of consciousness and control of bowel or bladder function [1]. Nearly 70 million people suffer from epilepsy worldwide, with India accounting for almost 12 million or about one-sixth of the global burden [2]. In addition to the huge public health burden, epilepsy also leads to social and cultural discrimination, impacting education, employment, marriage, and other essential social opportunities. People with epilepsy (PWE) face discrimination and stigma in most low and middle-income countries (LMICs) [3]. Further, the treatment gap of epilepsy in India has been reported to be between 22% and 95%, like other LMICs. This gap is found to be higher in rural areas and in women [3]. A higher treatment gap implies a higher disease burden, as a greater number of PWE would have access to no treatment or inadequate treatment.

Due to the smaller number of qualified neurologists in India, PWE may visit primary care physicians who are not trained in optimal management and lack thorough knowledge of the types of epilepsy and the use of AEDs [4]. Therefore, understanding the demographic profile, comorbidities, treatment modalities, and gaps in treatment patterns in PWE in India will help in early detection and primary prevention, reducing the treatment gap.

Previous studies undertaken in India have had a lower sample size. This study thus is the first of its kind in India that leverages electronic medical records as a primary source, to provide a detailed epidemiological profile, associated comorbidities, and risks of epilepsy. This study also analyzes and correlates the treatment pattern followed in clinical practice with the recommended treatment guidelines, such as the American Academy of Neurology (AAN) and GEMIND (Guidelines for the Management of Epilepsy in India), and evidence from the SANAD (Standard and New Anti-Epileptic Drugs) trial [5-8].

## Materials and methods

Data sources

Analysis was conducted on data from an Indian electronic software owned and administered by HealthPlix Technologies PRV. This software has been in operation since 2016 and fulfills the day-to-day operational needs of 16 medical specialties across 300+ cities in 20 states. This software captures longitudinal clinical information directly from the clinical encounter, including demographics, diagnosis, use of AEDs, underlying risk factors, tests, test results, procedures, functional status, and other data elements, which were then used to conduct the analysis.

Ethical compliance

The study was conducted as per the applicable national regulatory laws and guidelines as well as per the Helsinki Declaration. Patient confidentiality was always ensured since the study was performed using anonymized information only.

Ethics approval

The study protocol was approved on 11/11/2020 by Suraksha- Ethics Committee, Asian Institute of Medical Sciences, Mumbai, approval number: ECR/644/Inst/MH/2014/RR-17.

Study design

This retrospective observational study assessed EMR data of Indian patients diagnosed with epilepsy and those who had at least one anti-epileptic medication between January 2001 and December 2019. Although EMR was operational since 2016, the data captured in this study are from 2001. Past medical records of patients were imported into the current EMR to maintain their history. HealthPlix Technologies PRV identified the anti-epileptic medications that were prescribed to the patients by mapping the brand name on the prescriptions with the generic name.

Patients diagnosed with epilepsy in the database were included. The visit where the diagnosis was mentioned for the first time was considered as the baseline. Baseline analysis included demographics, type of epilepsy, risk factors, and choice of AED. Baseline patients were followed up for a visit at around six months to understand the treatment switch/add-on where data were available. Patients who had a confirmed history of epilepsy on EMR or prior to entry on EMR were excluded from this study.

**Figure 1:**
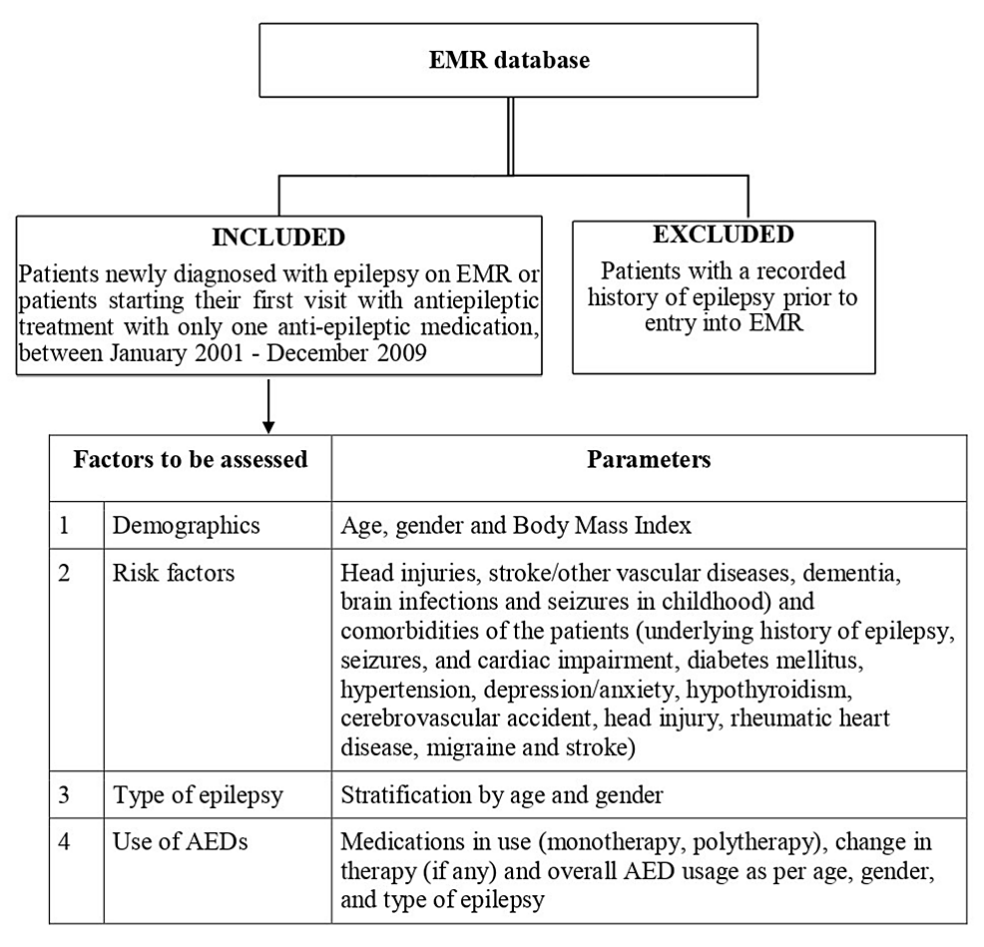
Overall study design with inclusion, exclusion criteria, and various parameters to be assessed

 For full details of the study design, including inclusion and exclusion criteria, refer to Figure 1.

Study endpoints

The primary endpoint was to assess the demographics, risk factors, co-morbidities, and type of epilepsy at baseline. The secondary endpoints were aimed at evaluating the usage of anti-epileptic drug(s) at baseline, treatment patterns, and choice of therapy (index AEDs or add-ons) based on age group, gender, type of epilepsy, and patient attributes. Furthermore, exploratory endpoints were assessed and analyzed for switch and add-ons for patients at the follow-up visit.

Assessments

This retrospective study used anonymous data extracted from the EMR. The EMR database captured longitudinal clinical information directly from the clinical encounter, including patient demographics, diagnosis, use of AEDs, underlying risk factors, tests, test results, procedures, functional status, and other data elements for patients receiving ambulatory care treatment at physicians’ offices across India. The EMR of all the patients satisfying the inclusion criteria of the study was collected. Inclusion criteria were mention of the diagnosis ‘Epilepsy’ or associated terms by the physician in the medical record. For further details, refer to Figure 1.

Statistical analysis

Pertinent retrospective data, relevant to the defined study objectives, were sourced from the EMR database and collated according to the study parameters using a pre-defined, templated data collection form. The collated and organized data were investigated to ensure the use of an accurate, reliable, consistent, and reproducible data set for subsequent statistical analyses. Any deviations in the data set, such as gaps, and missing and non-applicable data points were indicated and appropriately documented in the data collection form. The study sponsor had no direct access to the source EMR data. Central tendency and dispersion for continuous distributed data were evaluated and reported in terms of mean and standard deviation. Nominal data were reported in terms of numbers or/and percentages.

## Results

Primary endpoints

To assess demographics, risk factors, co-morbidities, and type(s) of epilepsy at baseline, a total of 12,424 patient EMRs were screened. Out of these, 6,318 patients with epilepsy met the inclusion criteria, as highlighted in Table 1, and data from these PWEs were evaluated. Seizures were classified as per the diagnosis made by the treating physician. Further, patients were categorized based on inclusion criteria into section A for new patients considering they had the diagnosis mentioned on any other visit except the first visit and B for patients with the first visit on EMR with one anti-epileptic medication. Since the history of these patients on the EMR is not available and they are on one anti-epileptic medication, they are considered at baseline.

**Table 1:** Enrolled patients based on inclusion criteria A and B

	Inclusion Criteria	Patient count (n)		Patient count (n)
A.	Patient diagnosed with seizure/epilepsy on HealthPlix EMR other than 1^st^ visit (Assumption: newly diagnosed patients)	1108	Visit 1 (Baseline)	1108
			Visit 2 (follow-up ≥ 6 months)	601
B.	Patient with only one anti-epileptic medication and epilepsy diagnosis on very 1st visit on the HealthPlix EMR	5210	Visit 1 (Baseline)	5210
			Visit 2 (follow-up ≥ 6 months)	2547

As presented in Table 2, males comprised 62% (n=3,908) of the study population and females represented the remaining 38% (n=2,409). The vast majority of patients were in the 18-55 (55%, n=3186) and >55 years’ age group (27% n=1655). Further, the majority of the patients were diagnosed with focal epilepsy (81%, n=5,141), followed by generalized epilepsy (9.5%, n =601) and unknown type (8.2%, n= 516).

**Table 2:** Patient demographics and vitals at visit 1 *98 patients in the age group <18 have no age information. One patient did not have age information in the age group >55 years. ** One patient did not have gender information. # Eight patients did not have geographical information. BMI: body mass index; SBP: systolic blood pressure; DBP: diastolic blood pressure

Parameter			Sub-Category	Inclusion Criteria A			Inclusion Criteria B				Units
				Patient count	Mean	SD		Patient count	Mean	SD	
Patients meeting inclusion criterion	Demographics	Age*	< 18 years	230	9.3	5.02		1148	10.3	4.94	Years
			18-55 years	519	34.9	11.36		2667	34.4	11.52	
			>55 years	347	69.04	33.05		1308	68.9	9.95	
		Gender**	Male	693	n/a			3215	n/a		Descriptive
			Female	415	n/a			1994	n/a		
		Geographic Distribution#	Class 1	287	n/a			1695	n/a		Descriptive
			Class 2-4	157	n/a			346	n/a		
			Metro	662	n/a			3162	n/a		
			Rural	n/a	n/a			1	n/a		
	Vitals at Baseline	Height	n/a	103	157.8	8.56		269	154.8	19.94	cm
		Weight	n/a	421	51.9	22.8		1934	52.5	22.65	Kg
		Body Mass Index (BMI)	BMI < 25	42	20.5	2.92		140	20.4	3.04	Kg/m2
			BMI 25 -29.9	20	28.1	1.38		71	27.01	1.45	
			BMI ≥ 30	15	32.6	2.01		41	34.9	6.6	
		Pulse (bpm)	n/a	251	83.4	13.82		1532	83.3	13	Bpm
		Blood Pressure	SBP	471	125.6	20.33		2628	124.5	18.18	mmHg
			DBP	471	80.8	11.85		2628	79.2	10.15	

**Figure 2:**
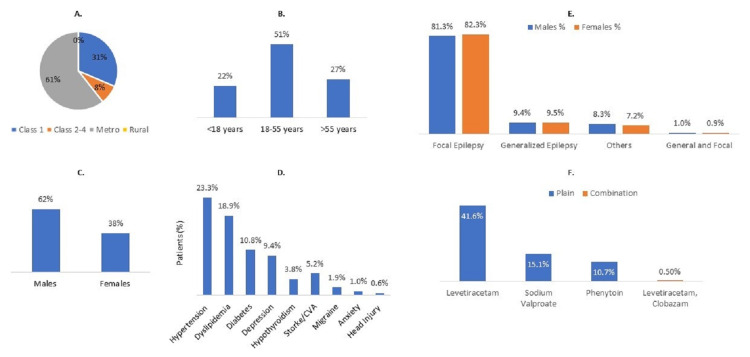
Overall patient demographics, vitals, epilepsy type, and AED usage A-C. Overall patient demographics by location, age, and gender. D. Major co-morbidities observed in the data collected for patients with epilepsy. E-F. Predominant types of baseline epilepsy and AED usage at baseline with top-three monotherapies and top combination therapy. AED: anti-epileptic drug

Refer to Figures 2A-2D for additional details. Data on comorbidities were available for 6,318 patients, the major comorbidity/medical conditions in PWE was hypertension (23.3%, n=1470), followed by diabetes mellitus (10.8%, n=683), and depression (9.4%, n=597). The most common risk factor was cerebrovascular accident (CVA)/stroke (5.3%, n=332) (the terms CVA and stroke have been used interchangeably) and migraine (1.9%, n=114), anxiety (1.0%, n=61), and head injury (0.6%, n=35).

Secondary and exploratory endpoints

As part of the study, we are also analyzing the choice of treatment determined from the EMRs with respect to different age groups, gender, and types of epilepsy. Exploratory endpoints included analyses to compare the treatment pattern followed in the database with recommended treatment guidelines. Guidelines are referred to pertaining to the treatment aspect of newly diagnosed epilepsy patients in terms of choice of drug at initiation and dosage versus observations seen in real-world practice.

Choice of treatment monotherapy vs polytherapy

Out of the total 6,318 patients who met the inclusion criteria, 6219 patients had age information and were further considered for analysis. We had 5899 (94.9%) patients on monotherapy and the rest were prescribed combination therapy.

Choice of treatment as per epilepsy type

Among the 6,219 patients, the preferred single-agent AED of choice in baseline was levetiracetam (41.6% n=2,589), followed by sodium valproate (15.1%, n=941), followed by phenytoin (10.7%, n=664) and in combination, the preferred AED agent was levetiracetam/clobazam (0.5%, n=33), as highlighted in Figure 2F.

A total of 5,141 (81.4%) had focal epilepsy. Levetiracetam was the most commonly prescribed AED in patients with focal epilepsy at visit 1 (41.0%, n=2108), followed by sodium valproate (13.7%, n=709), followed by phenytoin (11.7%, n=600). Refer to Table 3 for further details on AED usage and Table 4 on comorbidities for focal epilepsy patients. 

**Table 3:** AED usage in focal epilepsy based on age and gender AED: anti-epileptic drug

AEDs	<18 years			18-55 years			>55 years			Total AEDs
	F	M	Total	F	M	Total	F	M	Total	
Levetiracetam	146	160	307	506	586	1092	219	524	743	2142
Sodium Valproate	97	179	276	115	200	315	42	82	124	715
Phenytoin	9	31	40	81	193	274	90	197	287	601
Oxcarbazepine	50	81	131	101	152	253	25	48	73	457
Clobazam	38	66	104	66	71	137	8	23	31	272
Carbamazepine	9	18	27	60	77	137	19	37	56	220
Divalproex Sodium	13	28	41	37	40	77	15	18	33	151
Clonazepam	5	13	18	29	28	57	5	15	20	95
Lacosamide	5	6	11	19	23	42	4	21	25	78
Phenobarbitone	2	9	11	9	13	22	12	10	22	55
Topiramate	6	3	9	4	6	10	1	1	2	21
Levetiracetam, Clobazam	4	2	6	6	4	10	4	3	7	23
Total Focal Epilepsy	411	640	1052	1089	1499	2588	473	1028	1501	5141

**Table 4:** AED use in PWE with comorbidities AED: anti-epileptic drug; PWE: people with epilepsy

Comorbidity	Levetiracetam		Sodium Valproate		Phenytoin		Oxcarbazepine		Clobazam		Others AEDs	
	N	%	N	%	n	%	N	%	N	%	N	%
Hypertension	686	32.0%	116	16.2%	225	37.4%	68	14.9%	25	9.2%	169	26.3%
Diabetes	329	15.4%	45	6.3%	126	21.0%	25	5.5%	8	2.9%	59	9.2%
Depression	196	9.2%	58	8.1%	53	8.8%	43	9.4%	8	2.9%	95	14.8%
Hypothyroidism	84	3.9%	22	3.1%	35	5.8%	21	4.6%	8	2.9%	33	5.1%
Cerebrovascular accident	101	4.7%	10	1.4%	43	7.2%	7	1.5%	2	0.7%	10	1.6%
Stroke	72	3.4%	8	1.1%	13	2.2%	8	1.8%	4	1.5%	10	1.6%
Migraine	32	1.5%	13	1.8%	2	0.3%	7	1.5%	2	0.7%	37	5.8%
Anxiety	15	0.7%	7	1.0%	2	0.3%	3	0.7%	2	0.7%	15	2.3%
Head Injury	19	0.9%	0	0.0%	3	0.5%	1	0.2%	1	0.4%	1	0.2%
Total Patients	2142		715		601		457		272		643	

For 601 (9.5%) patients with generalized epilepsy, the most commonly prescribed agent at visit 1 was levetiracetam (44.3%, n=266), followed by sodium valproate (26.5%, n=162), followed by clobazam (5.6%, n=43). Sodium valproate had a much higher share in generalized epilepsy patients compared to focal epilepsy.

Choice of treatment in comparison to guidelines at baseline and at the follow-up visit

To determine the percentage of patients started on various monotherapy regimens at baseline and treatment patterns at the follow-up visit, data were collected for patients where dosage information was available for all visits. This was then compared to standard guidelines for the treatment and management of epilepsy, such as the AAN guideline and GEMIND, and evidence from the SANAD trial (Table 5) [5-7].

**Table 5:** Comparison of treatment guidelines vs real-world observations CBZ: carbamazepine; LEV: levetiracetam; LTG: lamotrigene; OXC: oxcarbazepine; PHT: phenytoin; PB: phenobarbital; VPA: sodium valproate; ZNS: zonsiamide

Guidelines	Guideline Recommendations for treatment of newly diagnosed Epilepsy	Real-world findings in patients with newly diagnosed Epilepsy
GEMIND Guidelines 2008	Treatment should be started with a single conventional anti-epileptic drug (AED monotherapy). Conventional AEDs are PHT, PB, CBZ, OXC, and VPA. Treatment should start with a low dose and gradually increase the dose until seizures are controlled	94.1% of patients were on monotherapy, 5.9% on polytherapy which is aligned with GEMIND, however, most prescribed monotherapy AEDs were LEV (newer AED), followed by VPA, and PHT Of the total 2930 monotherapy patients with a follow-up visit, 1316 patients had treatment for LEV, of which 1045 (80%) started at a 500 mg or higher dose and only 222 patients (17%) were prescribed a lower strength of 250 mg
AAN Guidelines 2018 for treatment of new-onset epilepsy	LTG (Level B evidence) should be considered to decrease seizure frequency in adults with new-onset focal epilepsy or unclassified tonic-clonic seizures (among newer agents). LEV, ZNS (Level C recommendation) to decrease seizure frequency	Of the total 6219 patients, only 36 patients (0.6%) were on LTG
SANAD II Trial	LTG should remain the first-line standard treatment for patients and the findings do not support the use of levetiracetam or zonisamide as first-line treatments for patients with focal epilepsy VPA to remain the first-line of treatment for patients with generalized epilepsy or seizures that are difficult to classify	For both patients with focal and generalized epilepsy, the treatment of choice is LEV followed by VPA VPA has a higher share in generalized epilepsy compared to focal, correlating to the trial outcome

Of the total 6,219 patients, 3,113 patients had data for the follow-up visit and these were considered for further analysis. Of these, 2,930 patients (94.1%) were on monotherapy. The rest were prescribed combination therapy. This is largely in line with GEMIND, which recommends initial antiseizure drug monotherapy for newly diagnosed patients.

GEMIND recommends the usage of a conventional anti-epileptic drug, at a lower dosage, while data suggest levetiracetam being the most prescribed agent at baseline, 1316 (44.9%) out of 2930 patients, with 500 mg being the most preferred strength (against the recommended 250 mg), 500 mg strength is followed by 250 mg. Levetiracetam is not among the list of conventional drugs as per GEMIND [6].

SANAD and AAN recommend the use of lamotrigine as first-line therapy for focal epilepsy patients as highlighted in Table 5 [5,7]. However, levetiracetam was most commonly prescribed AED for focal epilepsy patients at visit 1 (see Table 3).

For newly diagnosed generalized epilepsy, SANAD recommends the use of sodium valproate [8]. Data suggest levetiracetam was most prescribed for generalized epilepsy patients followed by valproate. SANAD does not recommend the usage of sodium valproate in women of childbearing age because of teratogenicity. While valproate usage is lower in females of childbearing age (115 out of 1089 or 10.6%) compared to all patients (715 out of 4830 or 14.8%), the number is substantial and in contrast with SANAD trial recommendations (see Table 5).

Table 3 depicts the choice of anti-epileptic drug, in focal epilepsy patients, based on age group, gender, type of epilepsy, whereas levetiracetam was the most commonly used AED in all the age groups, followed by sodium valproate, phenytoin, and oxcarbazepine

The most common comorbidity in PWE was hypertension, followed by diabetes mellitus and depression. Further, levetiracetam was again the most commonly used drug in patients with comorbidities (Table 4).

Treatment switch pattern for AED usage

Of the 1316 patients receiving levetiracetam, 33 patients switched over to other AEDs at visit 2 and another 138 patients received an add-on agent. The most common dose of levetiracetam at switch or add-on was 500 mg. The most prescribed switch/add-on agent was clobazam.

Levetiracetam was followed by sodium valproate as the most prescribed monotherapy at visit 1 (n=475) (Table 6). The highest dose at visit 1 was 600 mg twice daily (n=211). A total of 24 patients switched from sodium valproate monotherapy to other agents at visit 2. The most switched agents were levetiracetam (n=7) and clobazam (n=8). The highest dose of sodium valproate at the switch was 600 mg twice daily. A total of 59 patients had add-on therapy at visit 2 and the most common add-on agent was clobazam.

**Table 6:** AED monotherapy pattern of prescription AED: anti-epileptic drug

S no.	AED	Continued	87%	n=1145
1	Levetiracetam n= 1316	Switch/Add-on	13%	n=171
		1. Clobazam	4%	n=59
		2. Oxcarbazepine	2%	n=27
		3. Sodium Valproate	2%	n=25
2	Sodium valproate n= 475	Continued	83%	n=392
		Switch/Add-on	17%	n=83
		1. Clobazam	6%	n=29
		2. Levetiracetam	5%	n=23
		3. Oxcarbazepine	2%	n=9
3	Phenytoin n=308	Continued	84%	n=258
		Switch/Add-on	16%	n=50
		1. Levetiracetam	7%	n=21
		2. Clobazam	5%	n=14
4	Oxcarbazepine n=307	Continued	82%	n=251
		Switch/Add-on	18%	n=56
		1. Clobazam	7%	n=23
		2. Levetiracetam	7%	n=20
5	Clobazam n= 168	Continued	73%	n=123
		Switch/Add-on	27%	n=45
		1. Levetiracetam	8%	n=13
		2. Oxcarbazepine	5%	n=8
		3. Sodium Valproate	5%	n=8
6	Carbamazepine n=126	Continued	87%	n=109
		Switch/Add-on	13%	n=17
		1. Clobazam	7%	n=9
		2. Levetiracetam	2%	n=3

Overall, clobazam was the choice of AED in the second line (30.4%, n=143). However, patients who started on clobazam at baseline saw the highest switch/add-on to other agents at the follow-up visit.

## Discussion

The collected data suggested that the majority of the patients belonged to the age group 18-55 years (55%, n=3,186) with males and females being 62% and 38%, respectively. The most common underlying comorbidities were hypertension (23.3%, n=1470) and diabetes mellitus (10.8%, n=683). Further, the data also suggested that the majority of the patients were located in metros (61%, Refer to Figure 2A). A study conducted by Newale S et al. has demonstrated a higher prevalence of epilepsy in males versus females, similar to this study with diabetes and hypertension as the most common comorbidities reported [9]. Rosane B et al.’s study proposed hypertension as an independent risk factor for epilepsy, and both diabetes and hypertension might have an indirect effect on epilepsy causation, especially in the elderly, as these would predispose to a cerebrovascular accident (CVA)/stroke. The same age stratification was observed in this study, where 60% of patients with comorbidities (hypertension and diabetes) were in the elderly age group (>55 years) [10]. The reason for the difference in gender prevalence is not clear, however, a general observation reported by Alben S et al.’s study suggested that there was a higher incidence of focal epilepsy in males as compared to females [11]. The rate of occurrence of depression (9.4%, n=597) in PWE in the current study was also similar to the study conducted by Kirsten M F et al. [12]. One of the reasons for this high rate of depression might be social conditions or a higher frequency of seizures or non-availability or non-responsiveness to therapy resulting in poor quality of life. Also, a study conducted by Sirven JI et al. has suggested that the relationship between epilepsy and depression might be a two-way relationship [13].

The higher prevalence of focal epilepsy in the current study in 81% of the patients (n=5141) is similar to what has been reported by previous studies in India conducted by Santhosh S et al. and Divyani G et al. [4,14]. Some discrepancy can be explained by the fact that hospital‑based studies observed a higher frequency of focal epilepsy accounting for up to 80% of seizure types. However, in community‑based studies, generalized epilepsy was the more common type, with generalized tonic-clonic being the most common subtype. This could be attributed to the misrepresentation of the secondary generalization of focal epilepsy as primary generalized epilepsy in community settings [14].

AED usage data showed that the AED of choice at visit 1 was levetiracetam (41.6%, n=2589), followed by sodium valproate (15.1%, n=941) and phenytoin (10.7%, n=664) in monotherapy and levetiracetam/clobazam (0.5%, n=33) in polytherapy. Thus, at baseline, the majority of the patients (94.9%) patients were on monotherapy and the rest were on polytherapy. This data is in line with the study conducted by Alben et al. in India, which reported a higher preference for monotherapy; the study also suggested that the preference for monotherapy can be attributed to various pharmacotherapeutic and pharmacoeconomic benefits [11]. In terms of choice of AED, levetiracetam was the most commonly used AED as per our findings. This was consistent with findings from the studies conducted by Newale S et al. and Haroon A et al., which observed an increasing trend in the usage of newer AEDs, such as levetiracetam, as compared to conventional AEDs such as sodium valproate and phenytoin [9,15]. However, these findings were in contrast with the study conducted by Alben et al. in a tertiary care setting noted conventional drugs, such as carbamazepine and sodium valproate, had more preference while newer AEDs were used for add-on treatment [11].

Further, patients initiated on monotherapy were studied for follow-up visits to understand the treatment pattern as shown in Table 6. Overall, the treatment pattern observed, as shown in Table 6, does not necessarily seem to follow the recommended guidelines in terms of drugs of choice at initiation, add-on, switch. or dose at the switch. As per GEMIND, a switch or add-on should be attempted if higher doses of monotherapy do not provide desired relief [6]. As per our observation, the most common dose of levetiracetam at switch or add-on was 500 mg. Some of the most commonly prescribed AEDs at the initiation of therapy as per GEMIND were the conventional AEDs, namely, oxcarbazepine/carbamazepine, sodium valproate, phenytoin, and phenobarbitone. Conventional AEDs are relatively less expensive with well-known, long-term side effects [6]. However, we observed that levetiracetam was the most commonly used AED across all the studied age groups and in both genders. This is in accordance with a study conducted by Newale S et al. [9]. The other commonly used agents at initiation such as carbamazepine and phenytoin as shown in Table 6 were not part of the AAN recommendations for initial therapy [5].

Further, the SANAD trial recommends the use of sodium valproate as the first-line agent for new generalized epilepsy patients and does not recommend it in childbearing females. However, our data reported contrast findings where sodium valproate usage was followed by levetiracetam in terms of choice of treatment and usage was high among 18-55 years of age in generalized epilepsy patients [8]. This finding is similar to the study by Hyunmi K et al., which reported a noticeable proportion of women with epilepsy of childbearing age were treated with sodium valproate and topiramate despite known teratogenicity risks [16]. This study also explored reasons to understand why valproate was prescribed to women of childbearing age. They reported the frequent usage of sodium valproate in women who had comorbid mood or anxiety disorder. Valproate had been used for other non-epileptic conditions existing with epilepsy such as psychiatric disorders, headaches, or migraine. However, risk awareness should be increased among physicians for teratogenicity risks with sodium valproate [16].

Another important observation related to the prescription pattern was that clobazam was the most prescribed switch/add-on agent for patients who underwent a change in AED usage. This was consistent with the Indian study by Rupa J et al., which reported clobazam as an effective and well-tolerated add-on anti-epileptic drug [17].

## Conclusions

In conclusion, the data collected from this study are aligned but do not completely agree with GEMIND, AAN treatment guidelines, and SANAD. It affirms the treatment initiation with AED monotherapy; however, the treatment choices do not necessarily follow the recommended guidelines in terms of conventional AED drugs, at low strengths, at initiation. Some of the most commonly prescribed AEDs at the initiation of therapy as per GEMIND are the conventional AEDs, namely, oxcarbazepine, carbamazepine, sodium valproate, phenytoin, and phenobarbitone since those are less expensive and the side effects with long-term use are well known. Levetiracetam is the most commonly used agent for initiation therapy and is not a part of the GEMIND recommendations for initial therapy. However, later studies have established levetiracetam as a preferred treatment choice across epilepsy types. As per GEMIND, a switch or add-on should be attempted if higher doses of monotherapy are not providing the desired relief. Therefore, the treatment patterns we observed do not necessarily seem to follow the guideline,s as most of the cases of switches/add-ons were without an intervening increase in strength.

Our study shows that treatment decisions on initiation, switch, and add-on might be guided by personal experience of Indian health care providers (HCPs) with drugs and treatment being individualized as per patient profile. It is important to note that the access to electronic records reflects patient presentations at clinics and systems where electronic record-keeping occurs, which might inadvertently exclude patients from the most rural locations. In the future, a study capturing the sociodemographic distribution of epilepsy patterns and treatments could add significantly to this hypothesis. A longer follow-up period (> 6 months) would help strengthen the AED usage data presented here, but nonetheless, our study provides the first detailed insight into the prevalence of epilepsy and AED usage patterns across India.
